# Whipple's Disease (WD) Without Arthropathy in an Immunocompromised Patient

**DOI:** 10.7759/cureus.31659

**Published:** 2022-11-18

**Authors:** Ayham Khrais, Bing Han, Dhanasekaran Ramasamy, Shiva Kumar

**Affiliations:** 1 Medicine, Rutgers University New Jersey Medical School, Newark, USA; 2 Pathology, Cooperman Barnabas Medical Center, Livingston, USA; 3 Gastroenterology, Cooperman Barnabas Medical Center, Livingston, USA; 4 Gastroenterology and Hepatology, Digestive Disease Institute, Cleveland Clinic Abu Dhabi, Abu Dhabi, ARE

**Keywords:** tropheryma whipplei, hiv aids, immunocompromise, traveler's diarrhea, whipple's disease

## Abstract

Whipple’s disease (WD) is a rare disorder caused by the pathogen *Tropheryma whipplei (T. whipplei)*. We report a unique presentation of WD in which the patient did not exhibit arthralgia which is characteristic of this disease. A 67-year-old man with a history of chronic hepatitis B infection and human immunodeficiency virus (HIV) infection presented with weight loss, nausea, vomiting, and myalgia. Endoscopy demonstrated erythema in the gastric body, lymphangiectasia of the duodenum, and increased granularity of the terminal ileum. Mucosal biopsies revealed macrophages in the lamina propria with focal histiocytic aggregates throughout the small bowel and cecum, consistent with WD. Confirmatory *T. whipplei *polymerase chain reaction(PCR) testing was positive. WD is a rare diagnosis that must be considered in the differential diagnoses of patients presenting with unexplained nausea, vomiting, diarrhea, and anemia. Furthermore, in patients with HIV, the possibilities would also include opportunistic gastrointestinal pathogens. Classic WD is characterized by diarrhea, weight loss, abdominal pain, and extra-intestinal involvement manifesting as joint pain. We describe a case of WD occurring in a patient with HIV, without the disease's characteristic joint involvement.

## Introduction

The differential diagnoses of diarrhea, nausea, and vomiting in a patient with HIV are broad. Whipple’s disease (WD) is a rare systemic illness that can present similarly, can be difficult to diagnose in patients predisposed to opportunistic infections, and must be included in the differential diagnosis in this setting [1]. Furthermore, in patients with HIV, the possibilities would also include opportunistic gastrointestinal pathogens. Classic WD is characterized by diarrhea, weight loss, abdominal pain, and extra-intestinal involvement manifesting as joint pain, endocarditis, dementia, supranuclear gaze palsy, and mediastinal lymphadenopathy. Even though the definitive diagnosis of WD requires a tissue biopsy, immunohistochemistry can also be utilized as secondary confirmatory testing [2-6]. Treatment commonly includes two weeks of ceftriaxone followed by one year of suppressive therapy with trimethoprim-sulfamethoxazole, however, alternate antibiotic courses have been proposed to prevent disease relapse [2-3]. Relapse of WD can lead to further complications, including neurological involvement. We describe a case of WD made unique by the concomitant presence of HIV in the affected patient, and the lack of the disease's characteristic arthropathy.

This article was previously presented as a poster at the American College of Gastroenterology Conference on October 24, 2022. Abstracts accepted at the conference were published in a special supplement of the October 2022 issue of *The American Journal of Gastroenterology*.

## Case presentation

A 67-year-old African American male with a history of chronic hepatitis B infection and HIV (previous CD4 257 with undetectable viral load 4 months prior) on bictegravir, emtricitabine, and tenofovir presented to the emergency department with two months of weakness, poor nutritional intake, a 17-pound weight loss in one year, as well as four to five weeks of body aches, nausea and non-bloody, non-bilious vomiting and a two-week history of watery diarrhea. Medical history also included gastroesophageal reflux disease, hypertension, and hyperlipidemia. He was on no medications other than those taken for his HIV. On physical exam, he was awake, alert, and oriented, but appeared underweight. The abdomen was soft but tender to palpation throughout with no guarding.

**Figure 1:**
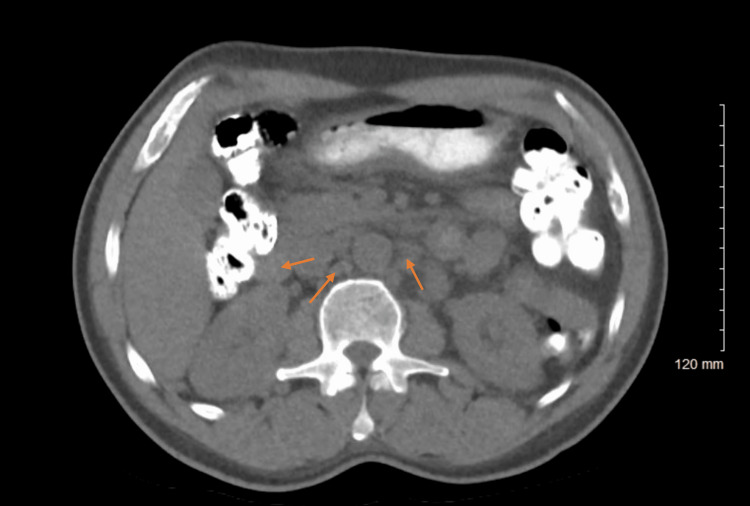
CT scan of the abdomen and pelvis demonstrating mesenteric and retroperitoneal lymphadenopathy (arrows).

Laboratory findings were significant for a hemoglobin of 8.6 g/dL, platelet count of 843 x 103/μL, white blood cell count of 8.8 x 103/μL, albumin 2.9 g/dL, and positive fecal occult blood test. Serum aminotransferases, alkaline phosphatase, and lipase levels were normal, while lactate dehydrogenase (LDH) was 161 units/L. HIV-1 RNA polymerase chain reaction (PCR) was negative, and the CD4 count was 257.1. CT scan of the abdomen and pelvis revealed extensive mesenteric and retroperitoneal lymphadenopathy, mildly increased from a scan two years prior (Figure 1). Biopsy of enlarged lymph nodes was significant for granulomatous lymphadenitis.

**Figure 2:**
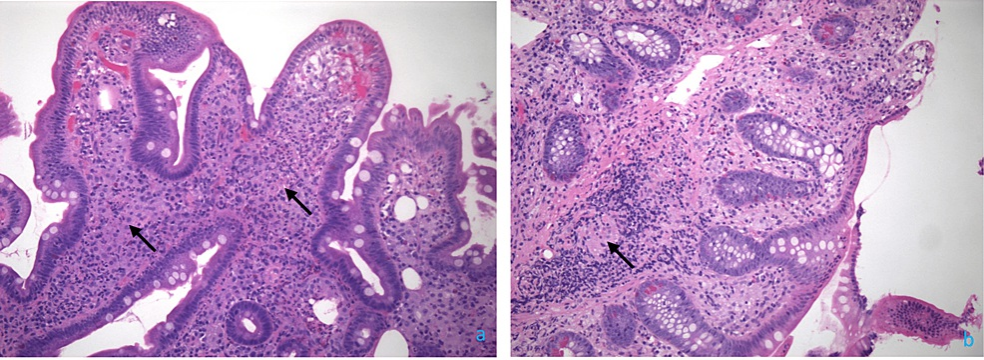
Hematoxylin and eosin staining of the biopsies from the duodenal bulb (a) and cecum (b) demonstrating expansion of lamina propria with abundant foamy macrophages (arrows) and neutrophils.

**Figure 3:**
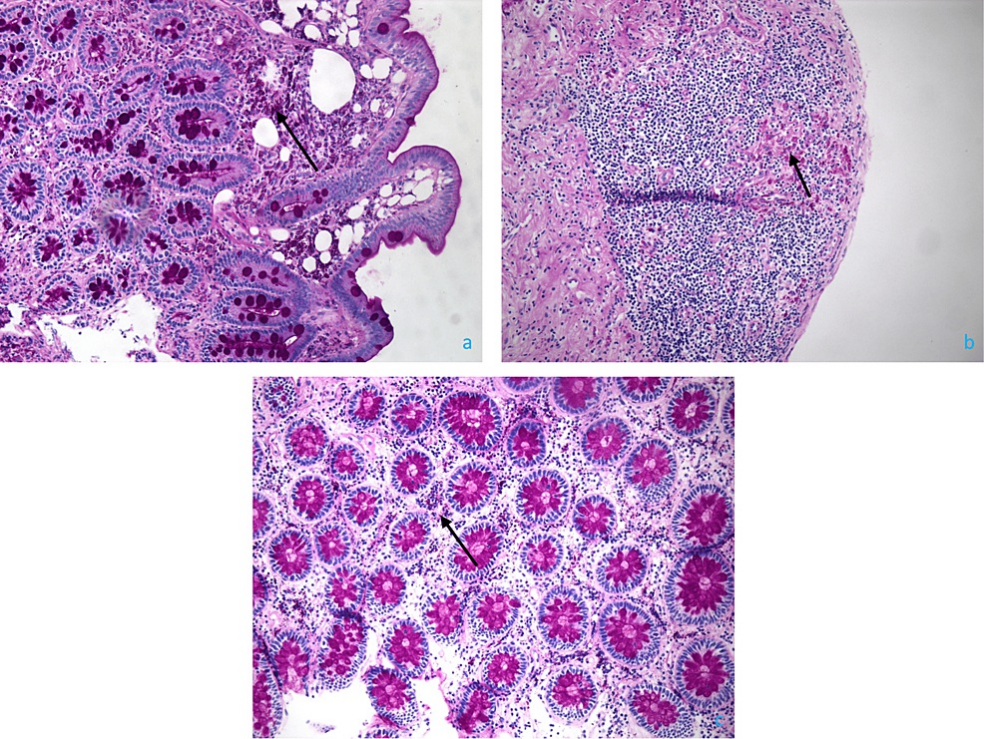
The PAS-stained macrophages (arrows) within the lamina propria of the duodenal bulb (a) and cecum (c), as well as the lymphoid aggregate in the cecum (b). PAS, periodic acid-Schiff

Upper endoscopy and colonoscopy demonstrated erythema in the gastric body, lymphangiectasia of the duodenum, and granularity of the terminal ileum. Pathology showed increased macrophages in the lamina propria with focal histiocytic aggregates throughout the small bowel and cecum (Figure 2), consistent with WD. Periodic-acid Schiff (PAS) staining was positive (Figure 3). Further testing for *Tropheryma whipplei* (*T. whipplei*) via PCR was positive. The patient was started on a 14-day course of 2 g IV ceftriaxone daily, followed by a one-year course of trimethoprim-sulfamethoxazole 160-800 mg twice a day.

## Discussion

Whipple’s disease has an incidence of 1 per 1,000,000, affecting middle-aged individuals most commonly in their 50s [2-3]. Classic WD is primarily a gastrointestinal disorder with multi-system involvement, caused by *Tropheryma whipplei* infection. *T. whipplei* is a Gram-positive rod of the Actinobacteria phylum [3, 6]. While the disease itself is rare, carriers of *T. whipplei* are fairly common, with a prevalence that varies from 1% to 36% [1, 5, 7-9]. Development of WD requires immunosuppression, resulting in a lack of macrophage and lymphocyte expression and action against the pathogen. A defect in phagocytosis results in a build-up of *T. whipplei*-containing macrophages in the mucosa of the small intestine [9]. This is demonstrated by the characteristic findings on small intestinal biopsy, consisting of PAS staining macrophages with inclusion bodies, found within the lamina propria [3-4].

Classic WD is characterized by diarrhea, weight loss, abdominal pain, and joint pain [2-4, 7]. Symptoms may take years to develop and to be diagnosed, due to their vague and chronic nature [8]. Arthritis, commonly preceding gastrointestinal (GI) symptoms, is chronic, intermittent, migratory, and affects multiple joints at once [4, 8]. The onset of GI symptoms usually follows arthralgia and consists of diarrhea and abdominal pain. Less common features of *T. whipplei* infection include mediastinal and mesenteric lymphadenopathy, endocarditis, and an interstitial lung disease-like presentation, which may be confused with sarcoidosis [2, 8]. Infection can result in central nervous system (CNS) disease as well, manifesting as dementia, seizures, ataxia, nystagmus, or supranuclear gaze palsy. Diagnosis of WD is difficult, as its presentation coincides with that of other, more common diseases. Therefore, a high index of suspicion for WD is warranted in the evaluation of unexplained diarrhea, weight loss, abdominal pain, and joint pain.

Definitive diagnosis is with a biopsy of the intestinal mucosa, demonstrating foamy macrophages with PAS (+) substance in the lamina propria [9]. Other histologic findings include fat particles in the lamina propria, lymphangiectasia, and dilated villi [4]. Alternatively, if biopsied tissue is PAS (-), one may utilize immunohistochemistry to identify anti-*T. whipplei* antigens, or *T. whipplei* PCR. Immunohistochemistry enables the diagnosis of WD before the appearance of PAS (+) macrophages in the lamina propria of the small bowel [3, 9].

Consensus on the treatment of WD varies across the literature. Many recommend an initial course of IV penicillin, IV ceftriaxone, or PO trimethoprim-sulfamethoxazole (TMP-SMX) for two to four weeks, followed by a one-year course of TMP-SMX [2-3, 9]. Long-term treatment with TMP-SMX, which penetrates the blood-brain barrier, prevents CNS relapse, which is common after a short course of antibiotics. Alternatively, a one-year course of doxycycline and hydroxychloroquine, followed by lifelong doxycycline therapy has been proposed, due to the bactericidal activity of these two agents against *T. whipplei* [5].

This patient’s presentation was unique due to the absence of arthralgia and co-existing HIV. In this context, WD may be overlooked due to the prevalence of more common pathogenic causes, including Microsporidium, Cryptosporidium, Giardia, and cytomegalovirus (CMV), as well as the possibility of GI disease due to the HIV infection itself [7, 10].

## Conclusions

Challenges in diagnosing WD are due to its rarity, presentation with non-specific symptoms, propensity for extra-intestinal involvement, and the chronic nature of symptoms. The physician must keep WD in the differential in patients with diarrhea, weight loss, abdominal pain and joint pain, even if their symptoms may be better explained by an identified chronic infection. Diagnosis with biopsy is the key, and prompt long-term therapy is necessary to avoid CNS involvement.
